# Right internal jugular vein distensibility appears to be a surrogate marker for inferior vena cava vein distensibility for evaluating fluid responsiveness

**DOI:** 10.5935/0103-507X.20150042

**Published:** 2015

**Authors:** Fabiano Broilo, Andre Meregalli, Gilberto Friedman

**Affiliations:** 1 Central Intensive Care Unit, Complexo Hospitalar Santa Casa - Porto Alegre (RS), Brazil.; 2 Postgraduate Program in Pneumological Sciences, Faculdade de Medicina, Universidade Federal do Rio Grande do Sul - Porto Alegre (RS), Brazil.

**Keywords:** Vena cava, inferior/ ultrasonography, Jugular veins/ ultrasonography, Fluid therapy, Respiration, artificial, Hemodynamics

## Abstract

**Objective:**

To investigate whether the respiratory variation of the inferior vena cava diameter (∆DIVC) and right internal jugular vein diameter (∆DRIJ) are correlated in mechanically ventilated patients.

**Methods:**

This study was a prospective clinical analysis in an intensive care unit at a university hospital. Thirty-nine mechanically ventilated patients with hemodynamic instability were included. ∆DIVC and ∆DRIJ were assessed by echography. Vein distensibility was calculated as the ratio of (A) Dmax - Dmin/Dmin and (B) Dmax - Dmin/ mean of Dmax - Dmin and expressed as a percentage.

**Results:**

∆DIVC and ∆DRIJ were correlated by both methods: (A) r = 0.34, p = 0.04 and (B) r = 0.51, p = 0.001. Using 18% for ∆DIVC, indicating fluid responsiveness by method (A), 16 patients were responders and 35 measurements showed agreement (weighted Kappa = 0.80). The area under the ROC curve was 0.951 (95%CI 0.830 - 0.993; cutoff = 18.92). Using 12% for ∆DIVC, indicating fluid responsiveness by method (B), 14 patients were responders and 32 measurements showed agreement (weighted Kappa = 0.65). The area under the ROC curve was 0.903 (95%CI 0.765 - 0.973; cut-off value = 11.86).

**Conclusion:**

The respiratory variation of the inferior vena cava and the right internal jugular veins are correlated and showed significant agreement. Evaluation of right internal jugular vein distensibility appears to be a surrogate marker for inferior vena cava vein distensibility for evaluating fluid responsiveness.

## Abstract (pt)

**Objetivo:**

Investigar se a variação respiratória no diâmetro da veia cava inferior (ΔDVCI) e no diâmetro da veia jugular interna direita (ΔDVJID) se correlacionam em pacientes submetidos à ventilação mecânica.

**Métodos:**

Estudo clínico prospectivo realizado em uma unidade de terapia intensiva de um hospital universitário. Foram incluídos 39 pacientes mecanicamente ventilados e com instabilidade hemodinâmica. Os valores da variação do diâmetro da veia cava inferior e da variação do diâmetro da veia jugular interna direita foram avaliados por meio de ecografia. A distensibilidade da veia foi calculada como a razão de (A) Dmin - Dmax/Dmin e (B) Dmax - Dmin/média de Dmax - Dmin, e expressa como porcentagem.

**Resultados:**

Com ambos os métodos, observou-se correlação entre a variação do diâmetro da veia cava inferior e a variação do diâmetro da veia jugular interna direita: (A) r = 0,34, p = 0,04 e (B) r = 0,51, p = 0,001. Utilizando o ponto de corte de 18% para indicar responsividade a fluidos na variação do diâmetro da veia cava inferior, pelo o método (A), 16 pacientes foram considerados responsivos e 35 medições mostraram concordância (Kappa ponderado = 0,80). A área sob a curva ROC foi de 0,951 (IC95% 0,830 - 0,993; valor de corte = 18,92). Usando 12% como ponto de corte para a variação do diâmetro da veia cava inferior para indicar capacidade de resposta a fluidos, pelo método (B), 14 pacientes foram responsivos e 32 medições mostraram concordância (Kappa ponderado = 0,65). A área sob a curva ROC foi de 0,903 (IC95% 0,765 - 0,973; valor de corte = 11,86).

**Conclusão:**

As variações respiratórias nas dimensões da veia cava inferior e da veia jugular interna direita se correlacionaram e mostraram concordância significativa. Avaliação da distensibilidade da veia jugular interna direita parece ser uma alternativa à distensibilidade da veia cava inferior para avaliar a responsividade a fluidos.

## INTRODUCTION

Circulatory failure is often the result of hypovolemia, which therefore must be corrected. Volume expansion improves the prognosis,^(1,2)^ whereas inappropriate use of vasoconstrictors leads to harmful tissue hypoperfusion.^(3,4)^ However, volume expansion may prove ineffective or even deleterious, by worsening pre-existing heart failure or by degrading gas exchange in a mechanically ventilated patient.^(5)^ Reliable tools for predicting the efficacy of volume expansion are therefore essential in critically ill patients. Several tools have proven sufficiently reliable, including minimally invasive measurements, such as variation in pulse pressure.^(6,7)^ The inferior vena cava (IVC) can be visualized by a subcostal approach. The IVC is a compliant blood vessel that is easily distended, especially in cases of hypovolemia.^(8,9)^ Mechanical ventilation induces cyclic variations in vena cava flow and diameter that are reflected in changes in blood flow within the time frame of a few heart beats.^(10,11)^ Those changes in flow have previously been shown to be accurate predictors of fluid responsiveness.^(5,12)^

However, IVC measurements are not possible in 10% to 15% of patients because of large body size, excessive bowel gas, or large amounts of intrathoracic air.^(13)^ It is well known that pressure and volume changes within the intrathoracic systemic venous compartment are reflected by the extrathoracic veins, such as in the extrathoracic internal jugular vein (IJV).^(14-16)^ Ultrasonography of IJV diameter has been studied in several studies to evaluate hypovolemia after blood donation.^(14,15)^ Recently, Guarracino et al. showed that IVJ distensibility accurately predicts volume responsiveness.^(17)^ They found that IJV distensibility more than 18% prior to volume challenge had an 80% sensitivity and 85% specificity in predicting response. The aim of our study was to test the hypothesis that respiratory changes in right internal jugular vein (RIJV) diameter in mechanically ventilated patients are similar to respiratory changes in IVC and therefore help to predict fluid responsiveness when visualization of the IVC is difficult.

## METHODS

### Patients

This prospective study was conducted over an 11-month period (February -December 2012) in the Central medical-surgical intensive care unit of the *Complexo Hospitalar Santa Casa*. Ventilated patients (> 18 years of age) were included when they presented with circulatory instability and required a rapid volume challenge according to the attending physician. The physician’s decision was based on the presence of clinical signs of acute circulatory failure (low blood pressure or urine output, tachycardia, mottling), and/or clinical signs of organ dysfunction (renal dysfunction, hyperlactacidemia).

Mechanical ventilation was performed in volume-controlled mode using a Servo Ventilator 300 (Siemens, Sweden). The study required perfect adaptation of the patient to the ventilator before starting the respiration cycle. All patients were in supine position with the head elevated to 30º and with ventilatory parameters adjusted to maintain a tidal volume of 6 - 10mL/kg and a positive end-expiratory pressure (PEEP) of 5 - 0cmH_2_O. The *Complexo Hospitalar Santa Casa* Research Ethics Committee approved this study (nº 38077214.1.0000.5335 - Plataforma Brasil) without the need for a consent form.

### Measurements

A single critical care physician with a certificate of ultrasound evaluation (basic competence),^(18)^ performed all of the ultrasound examinations (Siemens ACUSONX150, Korea). An associate critical care professor supervised both examinations. A two-dimensional echographic sector was used to visualize the inferior vena cava (sub-xyphoidal long-axis view), and its M-mode cursor was used to generate a time-motion record of the inferior vena cava diameter (DIVC) approximately 3 cm from the right atrium. Maximum and minimum DIVC values over a single respiratory cycle were collected. To visualize the RIJV a linear transducer was placed over the neck, using the sternocleidomastoid muscle as the external landmark; the IJV was evaluated just below the bifurcation of the sternal and clavicular heads of the muscle. To recognize the IJV, a gentle compression was used to differentiate it from the carotid artery. Thereafter, the probe pressure was relieved to avoid interfering with the IVJ diameters. The internal jugular vein on the transverse axis was recorded over a single respiratory cycle. Patients with evidence of jugular vein thrombosis or atrial fibrillation were excluded.

The distensibility index of inferior vena cava (ΔDIVC) and of the right internal jugular vein (ΔDRIJ), which reflect the increase in their diameters on inspiration, was calculated by two methods:

- Difference (Δ) between the maximum and the minimum diameter value/minimum diameter on expiration. Fluid responsiveness is defined when distensibility value for IVC is > 18%.^(9)^
- Difference (Δ) between the maximum and the minimum diameter value/mean of the two values. Fluid responsiveness is defined when distensibility value for IVC is > 12%.^(8)^

### Statistical analysis

For each parameter, the difference between values was compared using the independent sample *t* test. The correlation of parameters (crude data and after logarithmic transformation) was evaluated using the Pearson correlation test. P < 0.05 was regarded as statistically significant. The agreement between ΔDIVC and ΔDRIJ was assessed using weighted kappa measurement. To compare the predictive ability of ΔDRIJ to discriminate between fluid responders and non-responders, a computation of the area under the receiver operating characteristic (AUROC) curve was performed for both methods.

## RESULTS

A total of 46 patients were initially enrolled. Five patients were excluded because visualization of the IVC via ultrasound was technically difficult. Three of the patients had undergone laparotomy and the fourth was morbidly obese. Another 2 patients were excluded because RIJV was thrombosed on ultrasound. A total of 39 patients, 23 men (59%) and 16 women (41%), were included in the final analysis. Demographic characteristics, hemodynamic and ventilatory data are shown in table 1. Thirty patients were given norepinephrine and one was given dobutamine. No differences were observed in vena cava distensibility for central venous pressure (CVP), heart rate (HR), mean arterial pressure (MAP), Acute Physiology and Chronic Health Evaluation II (APACHE II) or Sequential Organ Failure Assessment (SOFA) scores between responders and non-responders by any method of calculation (Table 2).

**Table 1:** Demographic characteristics

**Parameters**	
Age (years)	64 ± 18
APACHE II	19 ± 10
SOFA	9 ± 3
Weight (kg)	75 ± 12
Height (cm)	168 ± 7
PBW (kg)	63 ± 8
fiO_2_	49 ± 13
Admission diagnosis*	
COPD	4
Systemic hypertension	25
Ischemic heart disease	12
Cerebral vascular disease	7
Cirrhosis	3
Chronic renal failure	9
Diabetes	16
Congestive heart failure	4
AIDS	1
Motive for a fluid challenge**	
Norepinephrine (≥ 2µg/kg/min)	17
CVP (≤ 8mmHg)	12
MAP (< 65mmHg)	10
Renal dysfunction	14
Arterial lactate (≥ 2.5mmol/L)	6

APACHE II - Acute Physiology and Chronic Health Evaluation; SOFA - sequential organ failure assessment; PBW - predicted body weight; FIO_2_ - inspiratory fraction of oxygen; COPD - chronic obstructive pulmonary disease; AIDS - acquired immunodeficiency syndrome; CVP - central venous pressure; MAP - mean arterial pressure.

* The total number of diagnoses is greater than 39 because one patient can have two or more diagnosis.

** The total number of motives for a fluid challenge is greater than 39 because according to the assistant doctor, there was more than one reason for a fluid bolus.

**Table 2:** Comparison of baseline values in responders and non-responders

	**Method A**		**Method B**		p value*
	**ΔDIVC cut-off 18%**		**ΔDIVC cut-off 12%**		
	**Responders (N = 16)**	**Non-responders (N = 23)**	**Responders (N = 14)**	**Non-responders** **(N = 25)**	**NS**
VT (ml/kg/PBW)	8.8 ± 1.8	8.1 ± 1.3	8.6 ± 1.7	8.3 ± 1.5	NS
MAP (mmHg)	73 ± 17	78 ± 15	72 ± 17	78 ± 15	NS
HR (beats/min)	105 ± 23	93 ± 15	107 ± 22	96 ± 116	NS
Norepinephrine# (µg/kg/min)	0.29 ± 0.25	0.37 ± 0.62	0.34 ± 0.25	0.34 ± 0.59	NS
	(N = 14)	(N = 16)	(N = 12)	(N = 18)	
CVP (mmHg)	14 ± 5	17 ± 8	15 ± 4	16 ± 8	NS
PEEP (cmH2O)	6.8 ± 2.3	7.4 ± 2.1	6.9 ± 2.4	7.2 ± 2.1	NS
ΔDRIJV	71 ± 83	13 ± 8	36 ± 29	9 ± 6	p < 0.002

ΔDIVC - distensibility of inferior vena cava; NS - not significant; VT - tidal volume; MAP - mean arterial pressure; HR - heart rate; CVP - central venous pressure; PEEP - positive end expiratory pressure; ΔDRIJV - distensibility of the right internal jugular vein.

* Independent sample *t*-test.

# 30 patients received an infusion of norepinephrine. The results are expressed as the mean ± standard deviation.

**Figure 1:**
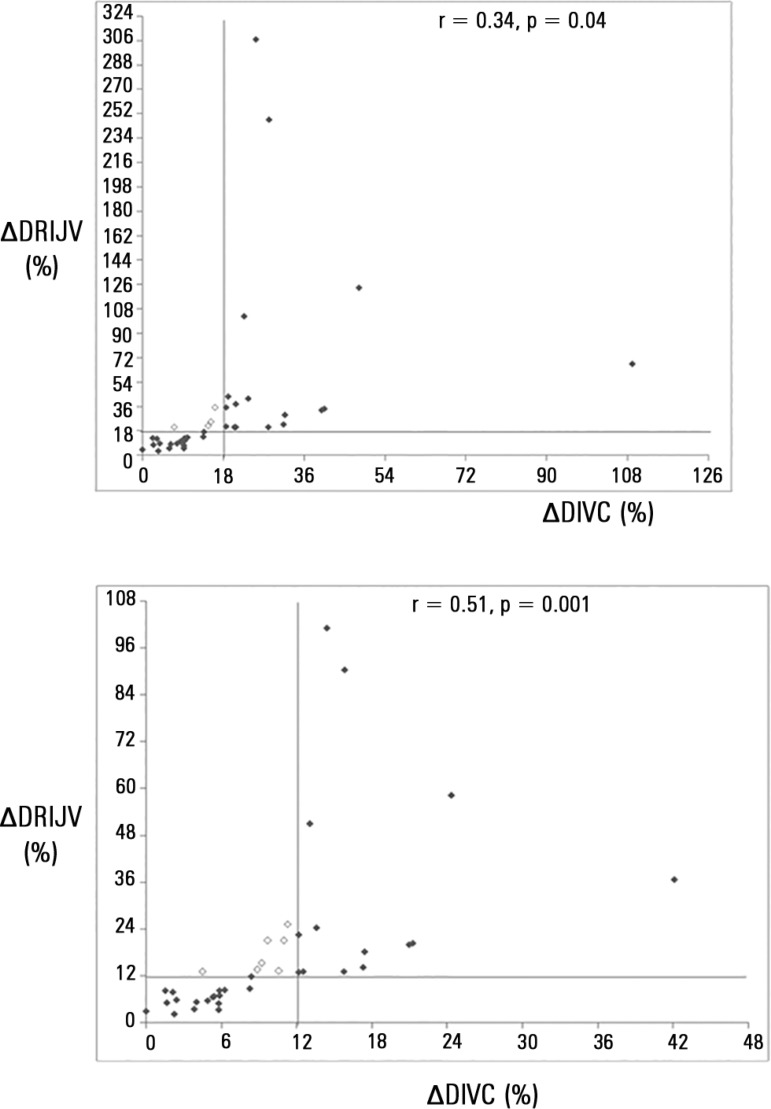
Distensibility of the inferior vena cava and of the right internal jugular vein are strongly correlated by method 1 (fluid responsiveness cut-off value: 18%) and method 2 (fluid responsiveness cut-off value: 12%). The empty points represent the points disagreeing. Pearson correlation test. ∆DIVC - distensibility of inferior vena cava; ∆DRIJV - distensibility right internal jugular vein.

**Figure 2:**
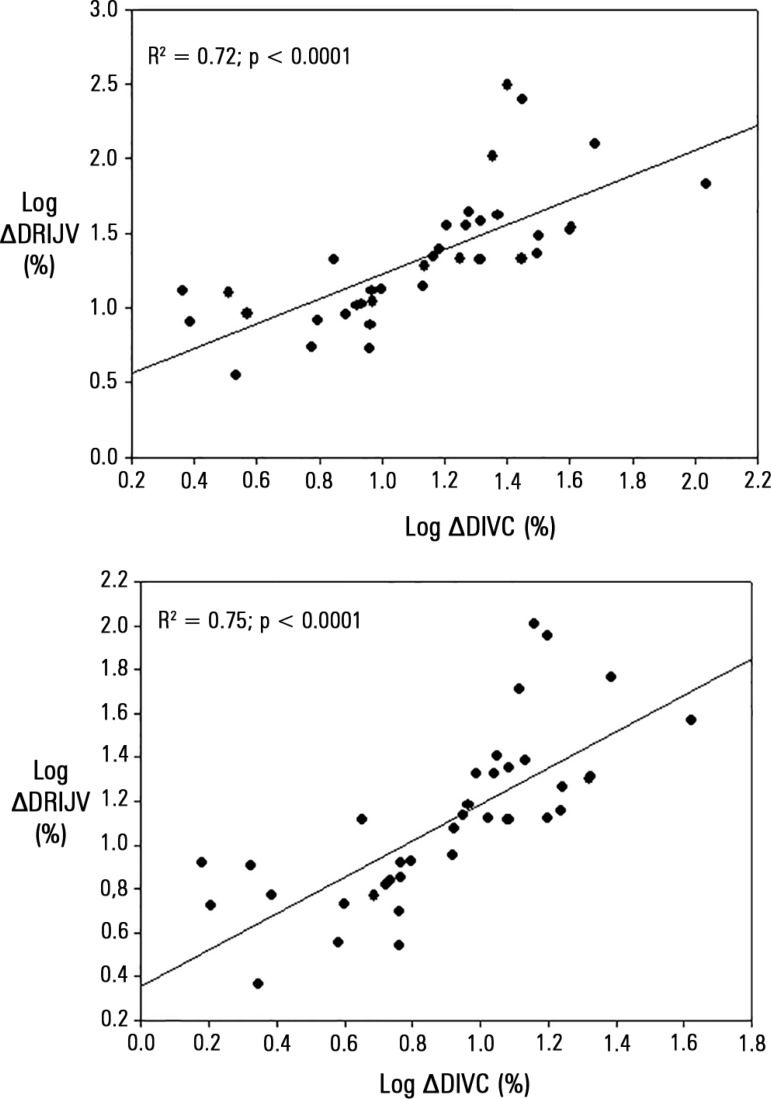
Pearson correlation after logarithmic transformation of the distensibility of the inferior vena cava and of the right internal jugular vein. ∆DIVC - distensibility of inferior vena cava; ∆DRIJV - distensibility right internal jugular vein.

The IVC anteroposterior diameter during inspiration was 21 ± 6mm, and during expiration was 18 ± 6mm (p < 0.0001). The inspiratory RIJV diameter was 11 ± 4mm and expiratory was 9 ± 4mm (p < 0.0001). ΔDIVC and ΔDRIJV were significantly correlated by both calculation methods (Figure 1). Correlations did not have a normal distribution, but log transformation revealed a highly significant correlation (Figure 2).

Using ΔDIVC of 18% as a cut-off value indicating fluid responsiveness for method A, 16 patients were responders and 35 measurements showed agreement (15 responders) with a very good weighted Kappa (k = 0.80). Using ΔDIVC of 12% as a cut-off value indicating fluid responsiveness for method B, 14 patients were responders and 32 measurements showed agreement (13 responders) with a good weighted Kappa (k = 0.65). Both methods agreed for 31 measurements.

**Figure 3:**
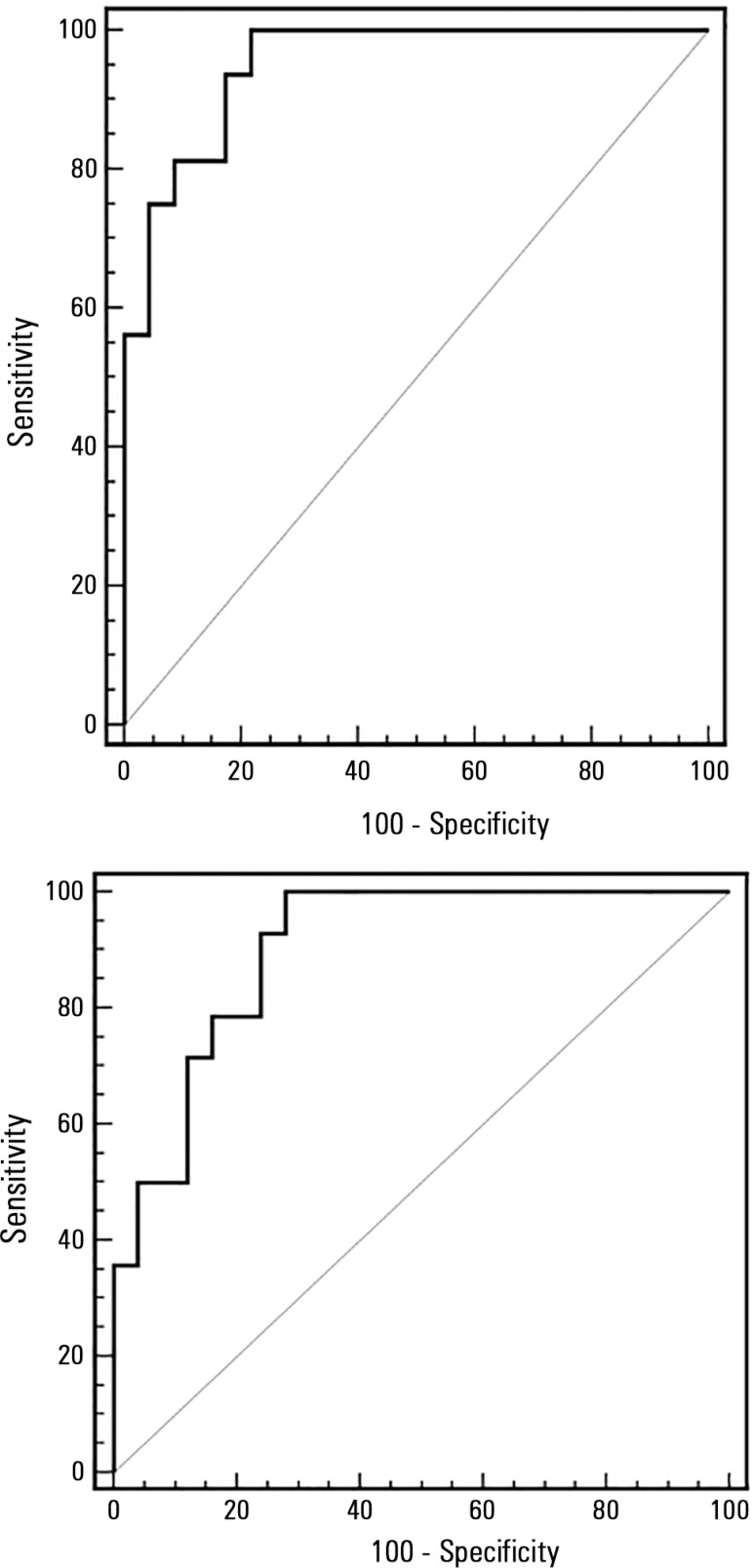
Receiver operating characteristic curve analysis of the right internal jugular vein distensibility index in predicting fluid responsiveness based on inferior vena cava distensibility values of 18% by method A and 12% by method B. The area under the ROC curve was 0.951 (95%CI 0.830 - 0.993) and 0.903 (95%CI 0.765 - 0.973), respectively. ∆DRIJV - distensibility right internal jugular vein; ∆DIVC - distensibility of inferior vena cava.

ΔDRIJV by method A showed an AUROC of 0.951 (95%CI 0.830 - 0.993) with a cut-off value of 18.92 (sensitivity 100%, specificity 78%). ΔDRIJV by method B showed an AUROC of 0.903 (95%CI 0.765 - 0.973) and a cut-off value of 11.86 (sensitivity 100, specificity 72%) (Figure 3).

## DISCUSSION

Our findings demonstrate that ultrasound evaluation of RIJV respiratory diameter changes can serve as a simple alternative or surrogate marker for IVC distensibility indexes in the evaluation of the appropriateness of volume expansion in mechanically ventilated patients.

Correcting hypovolemia is of paramount importance,^(1,2)^ but in mechanically ventilated patients, its correction should be guided to avoid ineffective or even deleterious volume expansion and worsening of the respiratory function.^(5)^ Mechanical ventilation induces cyclic variations in vena cava diameter that have been shown to be accurate predictors of fluid responsiveness.^(8,9,19)^ However, IVC measurements are often not possible.^(13)^

There are few studies investigating respiratory variations in RIJV diameter in the evaluation of hypovolemia or hemodynamic response to a fluid challenge, and these were conducted mainly in spontaneously breathing patients.^(14,15,17)^ During inspiration, the pressure inside the thorax increases more than the pressure outside the thorax. Therefore, the pressure gradient for venous return is reduced, the systemic venous return decreases, the volume of extrathoracic venous blood decreases, and hence the endoluminal diameter of distensible veins, such as the jugular vein increases.^(8,10,11)^ A greater decrease in venous return during insufflation may occur in a hypovolemic patient.

Our study demonstrated that the changes in IJV diameter during inspiration and expiration were significant. Similar findings were observed in several studies designed to evaluate IJV changes before and after blood donation^(14,15)^ or fluid challenge.^(17)^ However, in patients who are breathing spontaneously, the IJV collapse may be inexact.

In critically ill, mechanically ventilated patients, the subject is even less well studied. Recently, Guarracino et al. showed that IJV distensibility accurately predicts volume responsiveness.^(17)^ They measured cardiac output to calculate a cut-off of 18% with an 80% sensitivity and 85% specificity for predicting response. Thus, we compared the RIJV with IVC distensibility to predict fluid responsiveness, to explore the hypothesis that cyclic respiratory changes in both veins could be similar. In our population of mechanically ventilated patients with hemodynamic instability, we have shown that the IVC distensibility indexes and RIJV distensibility indexes agree and are well correlated. Taken together, despite the differences in study design, our findings agree with those of Guarracino et al.^(17)^ Although we have not evaluated volume expansion, the ΔIVC has been shown to be a good method for assessing fluid responsiveness in mechanically ventilated patients^(8,9)^ and our results show that ΔIVC and ΔRIJV correlate well.

In our study, approximately two thirds of the patients were non-responders. This finding is consistent with other studies designed to examine fluid responsiveness^(7-9,12,20-22)^ and strongly emphasize the need for parameters to help with selecting patients who might benefit from a volume load, avoiding ineffective or even deleterious volume expansion in non-responder patients.

Our study has several limitations. First, we have not evaluated fluid responsiveness after a fluid challenge to identify changes in cardiac output.^(17)^ Second, we did not evaluate changes in vein diameters before and after a fluid challenge. Third, we did not study conditions with high venous pressure or severe right heart failure that could reduce IVJ distensibility even in the presence of preload responsiveness. Fourth, one must be aware that ultrasound of the jugular vein should be performed by a skilled intensivist because even a little pressure could cause a great change in the cross-sectional image and diameter of the jugular vein during scanning. In patients with shock, venous scanning becomes even more difficult.^(18)^ Although all scans were performed by an intensivist certified in ultrasound, technical errors are possible. In addition, one could criticize that the scans were not repeated by another intensivist. Fifth, several patients were ventilated with low tidal volumes, which is a potential limitation for predicting fluid responsiveness.^(7)^ Although these limitations may introduce some bias, the consistency of the results implies improved external validity.

## CONCLUSION

In conclusion, internal jugular vein cyclic respiratory changes in diameter appear to be a possible surrogate for changes in inferior vena cava diameter in determining fluid responsiveness. Further studies should validate these findings by evaluating cardiac output after a fluid challenge in several clinical conditions.

## References

[1] Weil MH, Nishjima H (1978). Cardiac output in bacterial shock. Am J Med.

[2] Rivers E, Nguyen B, Havstad S, Ressler J, Muzzin A, Knoblich B, Peterson E, Tomlanovich M, Early Goal-Directed Therapy Collaborative Group (2001). Early goal-directed therapy in the treatment of severe sepsis and
septic shock. N Engl J Med.

[3] De Backer D, Biston P, Devriendt J, Madl C, Chochrad D, Aldecoa C, Brasseur A, Defrance P, Gottignies P, Vincent JL, SOAP II Investigators (2010). Comparison of dopamine and norepinephrine in the treatment of
shock. N Engl J Med.

[4] Murakawa K, Kobayashi A (1988). Effects of vasopressors on renal tissue gas tensions during
hemorrhagic shock in dogs. Crit Care Med.

[5] Pinsky MR, Teboul JL (2005). Assessment of indices of preload and volume
responsiveness. Curr Opin Crit Care.

[6] Michard F, Boussat S, Chemla D, Anguel N, Mercat A, Lecarpentier Y (2000). Relation between respiratory changes in arterial pulse pressure and
fluid responsiveness in septic patients with acute circulatory
failure. Am J Respir Crit Care Med.

[7] Oliveira-Costa CD, Friedman G, Vieira SR, Fialkow L (2012). Pulse pressure variation and prediction of fluid responsiveness in
patients ventilated with low tidal volumes. Clinics (Sao Paulo).

[8] Feissel M, Michard F, Faller JP, Teboul JL (2004). The respiratory variation in inferior vena cava diameter as a guide to
fluid therapy. Intensive Care Med.

[9] Barbier C, Loubières Y, Schmit C, Hayon J, Ricôme JL, Jardin F (2004). Respiratory changes in inferior vena cava diameter are helpful in
predicting fluid responsiveness in ventilated septic patients. Intensive Care Med.

[10] Morgan BC, Martin WE, Hornbein TF, Crawford EW, Guntheroth WG (1966). Hemodynamic effects of intermittent positive pressure
respiration. Anesthesiology.

[11] Natori H, Tamaki S, Kira S (1979). Ultrasonographic evaluation of ventilatory effect on inferior vena
caval configuration. Am Rev Respir Dis.

[12] Michard F, Teboul JL (2002). Predicting fluid responsiveness in ICU patients: a critical analysis
of the evidence. Chest.

[13] Nagdev AD, Merchant RC, Tirado-Gonzalez A, Sisson CA, Murphy MC (2010). Emergency department bedside ultrasonographic measurement of the caval
index for noninvasive determination of low central venous pressure. Ann Emerg Med.

[14] Akilli NB, Cander B, Dundar ZD, Koylu R (2012). A new parameter for the diagnosis of hemorrhagic shock: jugular
index. J Crit Care.

[15] Unluer EE, Kara PH (2013). Ultrasonography of jugular vein as a marker of hypovolemia in healthy
volunteers. Am J Emerg Med.

[16] Sankoff J, Zidulka A (2008). Non-invasive method for the rapid assessment of central venous
pressure: description and validation by a single examiner. West J Emerg Med.

[17] Guarracino F, Ferro B, Forfori F, Bertini P, Magliacane L, Pinsky MR (2014). Jugular vein distensibility predicts fluid responsiveness in septic
patients. Crit Care.

[18] Mayo PH, Beaulieu Y, Doelken P, Feller-Kopman D, Harrod C, Kaplan A (2009). American College of Chest Physicians/La Société de
Réanimation de Langue Française statement on competence in critical
care ultrasonography. Chest.

[19] Moretti R, Pizzi B (2010). Inferior vena cava distensibility as a predictor of fluid
responsiveness in patients with subarachnoid hemorrhage. Neurocrit Care.

[20] Huang CC, Fu JY, Hu HC, Kao KC, Chen NH, Hsieh MJ (2008). Prediction of fluid responsiveness in acute respiratory distress
syndrome patients ventilated with low tidal volume and high positive
end-expiratory pressure. Crit Care Med.

[21] Muller L, Bobbia X, Toumi M, Louart G, Molinari N, Ragonnet B, Quintard H, Leone M, Zoric L, Lefrant JY, AzuRea group (2012). Respiratory variations of inferior vena cava diameter to predict fluid
responsiveness in spontaneously breathing patients with acute circulatory failure:
need for a cautious use. Crit Care.

[22] Auler JO Jr, Galas FR, Sundin MR, Hajjar LA (2008). Arterial pulse pressure variation predicting fluid responsiveness in
critically ill patients. Shock.

